# 2729. Clinical characteristics, treatment and outcomes of infections among adult-onset immunodeficiency with anti-interferon (IFN)-γ autoantibodies patients: A single-center 10-year review from tertiary care hospital in Thailand

**DOI:** 10.1093/ofid/ofad500.2340

**Published:** 2023-11-27

**Authors:** Sorakai Wongpaiboonwatana, Thanarak Feungpunya, Jaruwan Diewsurin, Pornpit Treebupachatsakul

**Affiliations:** Buddhachinaraj hospital, Muang, Phitsanulok, Thailand; Buddhachinaraj hospital, Muang, Phitsanulok, Thailand; Buddhachinaraj hospital, Muang, Phitsanulok, Thailand; Buddhachinaraj hospital, Muang, Phitsanulok, Thailand

## Abstract

**Background:**

Adult-onset immunodeficiency (AOI) with anti-interferon (IFN)-γ autoantibodies is an acquired immunodeficiency syndrome increasingly recognized in East and Southeast Asia. Antibody against IFN- γ or its receptor is the main pathophysiology leading to opportunistic infections especially mycobacterial, dimorphic fungal or *Salmonella* infection. However, clinical information of infectious complications in this patient population remains scarce.

**Methods:**

A retrospective study was conducted in Buddhachinaraj hospital, Phitsanuloke, Thailand. We collected data on demographic, infectious complications, treatment and outcomes in patients with AOI with anti-IFN-γ autoantibodies who presented to our center between March 2013 and February 2023. All patients were diagnosed by elevation of anti-IFN- γ antibody level in serum more than 1.0.

**Results:**

A total of 35 patients. 54.3% were men with a median age of 58 years (IQR 14-63). Lymphadenopathy was the most common initial presentation (57%), followed by prolonged fever (40%), cough (11%) and skin lesions (11%). Lymph node involvement was found in 80% while 31% and 11% had intra-abdominal abscess and osteomyelitis.

Organisms were identified in 21 patients (60%). Mycobacterial infections were found in 19 patients (90%) which the main etiologic agent was *Mycobacterium abscessus* (43%). Six patients (28%) had evidence of mycobacterial infection but were unable to specified. *Histoplasma capsulatum* was found in 2 patients (9%). Another 14 patients (40%) were presumptive treated as non-tuberculous mycobacterium (NTM) infection without retrieving specific organism.

All patients were treated with antimicrobial agents according to identified causative organism. Fourteen patients with presumptive NTM infection were empirically treated with combination antibiotics. Median time of treatment was 24 months (IQR 12-35). Seven patients (20%) experienced relapse after antimicrobial discontinuation requiring retreatment. Three patients (6%) had died at median duration of 4 months from diagnosis.

**Conclusion:**

AOI with (IFN)-γ autoantibodies should be considered in patient whom suspected mycobacterium or fungal infection among non-HIV patients. Diagnosis and treatment are challenge and recurrent infection is common.

**Disclosures:**

**All Authors**: No reported disclosures

